# Supplementary material for: Physiological role for GABAA receptor desensitization in the induction of long-term potentiation at inhibitory synapses
Source: Nat Commun. 2021 Apr 9;12:2112. doi: 10.1038/s41467-021-22420-9 (PMC8035410; doi:10.1038/s41467-021-22420-9)
Supplement: Supplementary file 1 — Supplementary Information [file 41467_2021_22420_MOESM1_ESM.pdf]

# Physiological role for $GABA_A$ receptor desensitization in the induction of long-term potentiation at inhibitory synapses

Martin Field[1,2], Valentina Dorovykh[1], Philip Thomas[1] & Trevor G Smart[1,*]

[1] Department of Neuroscience, Physiology & Pharmacology, UCL, Gower Street, London, United Kingdom, WC1E 6BT
[2] Current affiliation: Department of Pharmacology, University of Oxford, Oxford, United Kingdom, OX1 3QT

*Corresponding author – Trevor G Smart (t.smart@ucl.ac.uk)

Supplementary information

## Supplementary figure 1

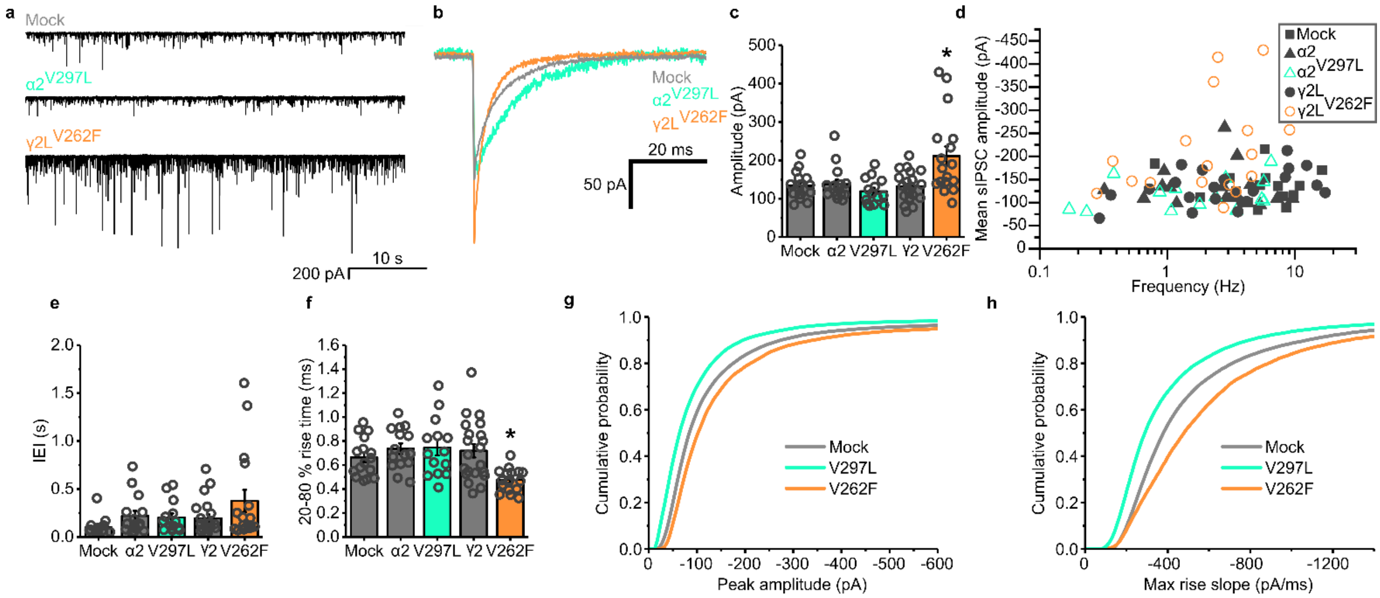


**Supplementary Figure 1. $\gamma 2^{V262F}$ expression increases sIPSC amplitudes at physiological temperature. a** Representative sIPSCs recorded at 37°C from voltage clamped neurons expressing the indicated constructs (Mock transfection, black; $\alpha 2^{V297L}$, green; $\gamma 2^{V262F}$, orange). **b** Average sIPSC unscaled waveforms and **c**, summary bar graph for mean sIPSC amplitudes: n = 16 (mock transfected), 15, 14, 21, and 17 neurons, for each construct as they appear on the abscissa (one-way ANOVA: $F_{(4, 78)} = 6.67$, p = 0.00011; Tukey test (mock vs $\alpha 2^{V297L}$): p = 0.96; Tukey test (mock vs $\gamma 2l^{V262F}$): p = 0.0022). **d** Mean sIPSC amplitude plotted against average sIPSC frequency for each construct (see symbol key). **e** Median IEI for all cells (one-way ANOVA: $F_{(4,78)} = 3.49$, p = 0.011; Tukey test (mock, V262F): p = 0.0099). **f** Mean 20-80% rise times for all cells (one-way ANOVA: $F_{(4,78)} = 5.51$, p = 0.00059 Tukey test ($\gamma 2^{V262F}$): p = 0.0024). **g** Mean cumulative probability distributions of the amplitudes and **h** maximum rate of rise slopes of all sIPSCs recorded. n = 54111 events from 16 cells (Mock), 24531 events from 14 cells ($a2^{V297L}$), and 24663 events from 17 cells ($g2L^{V262F}$). Source data are provided as a Source data file for Supplementary Fig 1.

## Supplementary Table 1: mutagenesis details

| Mutation | Forward primer | Reverse primer |
|---|---|---|
| $\alpha2^{V297L}$ | CTGTTCTCTGCCCTAATTGAATTTGCA | AAACGCATAACAAACAGCTATAAACCAGTCC |
| | References | |
| $\alpha1^{V296L}$ | Gielen, M., Thomas, P., and Smart, T.G. (2015). The desensitization gate of inhibitory Cys-loop receptors. *Nature Communications* **6**, 6829, doi.org/10.1038/ncomms7829. | |
| $\gamma2L^{V262F}$ | Gielen, M., Thomas, P., and Smart, T.G. (2015). The desensitization gate of inhibitory Cys-loop receptors. *Nature Communications* **6**, 6829, doi.org/10.1038/ncomms7829. | |
| $\gamma2L^{S327A}$ | Krishek, B. J. et al. Regulation of $GABA_A$ receptor function by protein kinase C phosphorylation. *Neuron* **12**, 1081–1095 (1994). | |

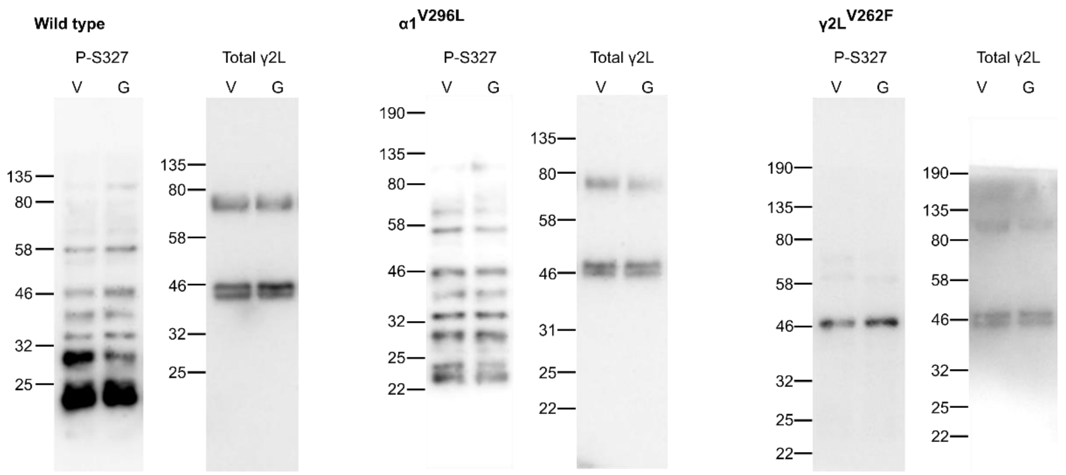


**Whole blots for the panels in Fig. 8j.** All molecular weight markers are given in kDa.
