# Supplementary material for: Physiological role for GABAA receptor desensitization in the induction of long-term potentiation at inhibitory synapses
Source: Nat Commun. 2021 Apr 9;12:2112. doi: 10.1038/s41467-021-22420-9 (PMC8035410; doi:10.1038/s41467-021-22420-9)
Supplement: Supplementary file 2 — Reporting Summary [file 41467_2021_22420_MOESM2_ESM.pdf]

nature research

Corresponding author(s): Trevor G Smart

Last updated by author(s): Feb 9, 2021

Reporting Summary

Nature Research wishes to improve the reproducibility of the work that we publish. This form provides structure for consistency and transparency in reporting. For further information on Nature Research policies, seeAuthors & Referees and theEditorial Policy Checklist .

Statistics

For all statistical analyses, confirm that the following items are present in the figure legend, table legend, main text, or Methods section.

n/a	Confirmed	
☐	☒	The exact sample size (*n*) for each experimental group/condition, given as a discrete number and unit of measurement
☐	☒	A statement on whether measurements were taken from distinct samples or whether the same sample was measured repeatedly
☐	☒	The statistical test(s) used AND whether they are one- or two-sided<br>*Only common tests should be described solely by name; describe more complex techniques in the Methods section.*
☒	☐	A description of all covariates tested
☐	☒	A description of any assumptions or corrections, such as tests of normality and adjustment for multiple comparisons
☐	☒	A full description of the statistical parameters including central tendency (e.g. means) or other basic estimates (e.g. regression coefficient) AND variation (e.g. standard deviation) or associated estimates of uncertainty (e.g. confidence intervals)
☐	☒	For null hypothesis testing, the test statistic (e.g. *F*, *t*, *r*) with confidence intervals, effect sizes, degrees of freedom and *P* value noted<br>*Give P values as exact values whenever suitable.*
☒	☐	For Bayesian analysis, information on the choice of priors and Markov chain Monte Carlo settings
☒	☐	For hierarchical and complex designs, identification of the appropriate level for tests and full reporting of outcomes
☒	☐	Estimates of effect sizes (e.g. Cohen's *d*, Pearson's *r*), indicating how they were calculated

Our web collection on statistics for biologists contains articles on many of the points above.

Software and code

Policy information about availability of computer code

Data collection	Clampex Ver 10.2 - for patch clamp recordings
Data analysis	Clampfit ver 10.7.0.3 (Molecular devices) - for analysing electrophysiological data.<br>Originpro 2018 (OriginLab Corporation) - for generating graphs and statistical tests.<br>WinWCP Ver 5.2.3 - for non-stationary fluctuation analysis.<br>R version 3.6.2 - for PCA analysis<br>EMMIXskew package ver 1.0.3 - for cluster analysis<br>GelAnalyser 2010a ver 19.1 - for gel analysis

For manuscripts utilizing custom algorithms or software that are central to the research but not yet described in published literature, software must be made available to editors/reviewers. We strongly encourage code deposition in a community repository (e.g. GitHub). See the Nature Research guidelines for submitting code & software for further information.

Data

Policy information about availability of data

All manuscripts must include a data availability statement. This statement should provide the following information, where applicable:

- Accession codes, unique identifiers, or web links for publicly available datasets
- A list of figures that have associated raw data
- A description of any restrictions on data availability

The datasets generated and analysed during this current study are either included in this published article (and its supplementary information files) and can be made available from the authors on reasonable request.

Field-specific reporting

Please select the one below that is the best fit for your research. If you are not sure, read the appropriate sections before making your selection.

- [x] Life sciences
- [ ] Behavioural & social sciences
- [ ] Ecological, evolutionary & environmental sciences

For a reference copy of the document with all sections, see nature.com/documents/nr-reporting-summary-flat.pdf

Life sciences study design

All studies must disclose on these points even when the disclosure is negative.

Sample size	Sample sizes for our experiments were based on many previous studies where adequate statistical power was achieved at P= 0.05.
Data exclusions	No data were excluded from this study
Replication	Number of replicates for each experiment is stated in the figure legends.
Randomization	Systematic randomization was not used in this study as it is impractical for such in vitro experiments that invariably have the control and drug-test designed into the same experiment
Blinding	Investigators were not blind to study conditions since this is difficult to achieve when the electrophysiological signals are so easily recognised, ie, GABA-activated currents

Reporting for specific materials, systems and methods

We require information from authors about some types of materials, experimental systems and methods used in many studies. Here, indicate whether each material, system or method listed is relevant to your study. If you are not sure if a list item applies to your research, read the appropriate section before selecting a response.

Materials & experimental systems

n/a	Involved in the study	
☐	☒	Antibodies
☐	☒	Eukaryotic cell lines
☒	☐	Palaeontology
☐	☒	Animals and other organisms
☒	☐	Human research participants
☒	☐	Clinical data

Methods

n/a	Involved in the study	
☒	☐	ChIP-seq
☒	☐	Flow cytometry
☒	☐	MRI-based neuroimaging

Antibodies

Antibodies used

anti-γ2 phospho-S327, ab73183 (Abcam), 1:1000
anti-γ2: Alomone, 1:500, AGA-005
HRP-conjugate anti-rabbit, 1:10000, Rockland

Validation

AGA-005: Sequence used to generate the antibody is identical in rat and mouse
Refs:
Mouse brain lysate (1:300).
Du, Z. et al. (2016) Neuroscience 329, 363.
HEK 293 transfected cells.
Wang, J. et al. (2016) Sci. Rep. 6, 35294.
HEK 293T transfected cells (1:100).
Warner, T.A. et al. (2016) Hum. Mol. Genet. 25, 3192.
Mouse brain lysate.
Lo, W.Y. et al. (2014) Neurochem. Res. 39, 1088.
Mouse brain lysate (1:200).
Vlachos, A. et al. (2013) Cereb. Cortex 23, 2700.
ab73183: verified by manufacturer with immunoblotting, blocked by immunising peptide and lambda phosphatase
Refs:
immunocytochemistry: Fricke et al. (2019) Cell Reports, 29, 671

Eukaryotic cell lines

Policy information about cell lines

Cell line source(s)	Human embryonic kidney (HEK) - 293 cells (CRL-1573), ATCC Cell lines, Virginia, USA
Authentication	Rely on authentication by ATCC - we check cell physiology routinely
Mycoplasma contamination	Cells not tested for mycoplasma infection during this study.
Commonly misidentified lines (See ICLAC register)	No commonly misidentified cell lines were used in the study.

Animals and other organisms

Policy information about studies involving animals; ARRIVE guidelines recommended for reporting animal research

Laboratory animals	Hippocampi from both male and female Sprague-Dawley E18 pups were used to generate the neuronal cultures used in the study.
Wild animals	No wild animals were used in this study
Field-collected samples	No field-collected samples were used in this study
Ethics oversight	Not applicable

Note that full information on the approval of the study protocol must also be provided in the manuscript.
